# A RelA(p65) Thr505 phospho-site mutation reveals an important mechanism regulating NF-κB-dependent liver regeneration and cancer

**DOI:** 10.1038/onc.2015.526

**Published:** 2016-02-08

**Authors:** A Moles, J A Butterworth, A Sanchez, J E Hunter, J Leslie, H Sellier, D Tiniakos, S J Cockell, D A Mann, F Oakley, N D Perkins

**Affiliations:** 1 Institute of Cellular Medicine, Faculty of Medical Sciences, Newcastle University, Newcastle Upon Tyne, UK; 2 Institute for Cell and Molecular Biosciences (ICaMB), Faculty of Medical Sciences, Newcastle University, Newcastle Upon Tyne, UK; 3 Bioinformatics Support Unit, Faculty of Medical Sciences, Newcastle University, Newcastle Upon Tyne, UK

## Abstract

Post-translational modifications of nuclear factor (NF)-κB subunits provide a mechanism to differentially regulate their activity in response to the many stimuli that induce this pathway. However, the physiological significance of these modifications is largely unknown, and it remains unclear if these have a critical role in the normal and pathological functions of NF-κB *in vivo*. Among these, phosphorylation of the RelA(p65) Thr505 residue has been described as an important regulator of NF-κB activity in cell lines, but its physiological significance was not known. Therefore, to learn more about the role of this pathway *in vivo*, we generated a knockin mouse with a RelA T505A mutation. Unlike RelA knockout mice, the RelA T505A mice develop normally but exhibit aberrant hepatocyte proliferation following liver partial hepatectomy or damage resulting from carbon tetrachloride (CCl_4_) treatment. Consistent with these effects, RelA T505A mice exhibit earlier onset of cancer in the *N*-nitrosodiethylamine model of hepatocellular carcinoma. These data reveal a critical pathway controlling NF-κB function in the liver that acts to suppress the tumour-promoting activities of RelA.

## Introduction

The nuclear factor (NF)-κB transcription factor family are important regulators of immune and inflammatory responses.^1^ Furthermore, aberrantly active NF-κB is associated with many human diseases, including cancer.^2, 3, 4^ While the pathways leading to nuclear translocation of NF-κB provide a primary level of regulation of its activity, NF-κB subunits are also subject to a wide range of post-translational modifications (PTMs). These can contribute to the control of nuclear translocation, but also have major effects on the function of NF-κB subunits, including induction of protein degradation, enhancement of DNA binding, as well as transcriptional effects, such as stimulation or inhibition of coactivator/corepressor binding.^5, 6, 7^ PTMs provide a mechanism to differentially regulate the transcriptional activity of NF-κB in response to diverse stimuli in different cell types.^4^

The C-terminal RelA (p65) transactivation domain is responsible for mediating most of this NF-κB subunit's transcriptional effects and contains a number of highly conserved known and putative phosphorylation sites.^6^ It has been suggested that different patterns of phosphorylation of these sites can control the specificity of NF-κB target gene activation or repression.^4, 8^ This can result in different context-dependent functions of NF-κB. A good example of this is the RelA-dependent anti-apoptotic effects seen following treatment with inflammatory stimuli, such as tumor necrosis factor (TNF)-α that contrast with pro-apoptotic effects following treatment with some DNA damage agents.^9, 10, 11, 12, 13, 14^ Understanding the mechanistic basis for these contrasting effects has important implications for the role of NF-κB in tumorigenesis and the response to cancer therapies.^4^

**Figure 1:**
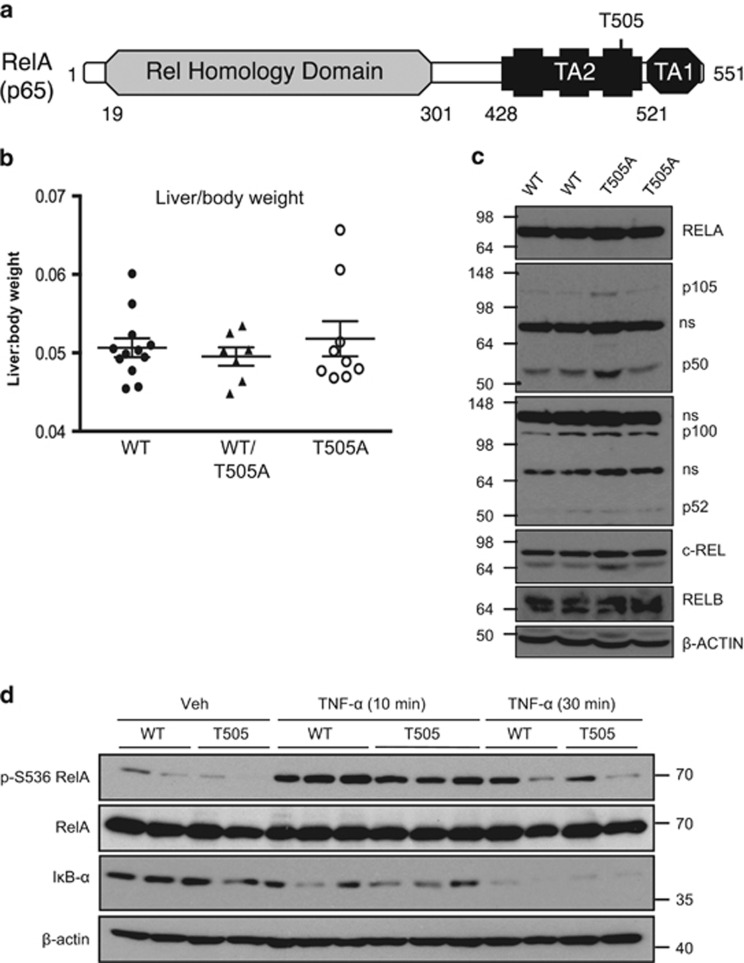
The phenotype of RelA T505A mice. (**a**) Schematic diagram of RelA showing the location of the Thr505 residue in transactivation domain 2. (**b**) Liver body weight ratio of wild type (WT), WT/T505A and T505A 12-week-old littermate mice. WT, *n=*4 males, 8 females; WT/T505A, *n=*2 males, 5 females; T505A, *n=*4 males, 5 females. Data represent mean±s.e.m. (**c**) Western blots showing relative levels of NF-κB subunits in WT and RelA T505A mice. (**d**) Western blot analysis of phospho-S536 RelA, RelA, IκB-α and β-actin from WT and RelA T505A hepatocytes. Hepatocytes were cultured in 0% FBS media and treated with 50 ng/ml of TNF-α for 10 or 30 min before preparation of cell lysates. Lanes represent independent experimental replicates from a single hepatocyte isolation. These results are representative of data from three separate hepatocyte preparations. In this and subsequent experiments, all mice were designated to an experimental group dependent on their strain. NS, not significant.

Among these pathways, phosphorylation of the RelA(p65) Thr505 residue (Figure 1a) provides a mechanism of crosstalk between NF-κB signalling and DNA replication stress. Our group had previously observed that phosphorylation of RelA at Thr505 by Chk1 correlated with a pro-apoptotic form of NF-κB in response to cisplatin treatment and following induction of the ARF tumour suppressor.^11, 12, 15, 16^ Additionally, we found that RelA Thr505 phosphorylation negatively regulates diverse cellular functions, such as proliferation, migration and autophagy.^12^ These data suggested that Thr505 mutation would remove the functions of RelA associated with tumour suppressor characteristics, resulting in a form of RelA with enhanced tumour-promoting activities, including the abilities to promote cell survival and proliferation. However, in common with many other NF-κB subunit PTMs, *in vivo* evidence for such an important functional role has been lacking. Therefore, to address this, we have generated a knockin mouse mutant where the RelA Thr505 residue has been changed to alanine. We hypothesised that selectively removing a specific mechanism of regulating NF-κB would provide important functional insights not seen with whole-gene deletion, where due to the pleiotropic functions of this pathway subtler effects are masked. In this report, we have investigated the effect of this mutation on the response to liver injury and chemically induced hepatocellular carcinoma, both processes requiring NF-κB activity.^17, 18, 19, 20, 21^ We find that mutation of RelA Thr505 leads to an aberrant proliferative response after liver injury and earlier onset of hepatocellular carcinoma, revealing an important mechanism normally acting to suppress the tumour-promoting functions of NF-κB.

## Results

### Creation of the RelA T505A knockin mouse

Although studies based on cell lines have indicated an important regulatory role for RelA Thr505 phosphorylation, the significance of this modification *in vivo* is not known. Therefore, to answer this question, a C57Bl/6 mouse was generated where this residue was mutated to alanine. By contrast with RelA knockout mice, which die in utero as a result of TNF-induced liver apoptosis,^9^ RelA T505A mice were healthy with no overt effects on ageing or mortality up to 18 months of age (not shown). The absence of an effect on viability is consistent with previous observations from this group, where mutation of this site was determined not to affect TNF-induced NF-κB activity or survival.^12^ Analysis of the organs from 12-week-old littermates revealed no statistically significant differences in the weight of the liver, heart and kidneys (Figure 1b; Supplementary Figures S1A and B). Furthermore, no differences in the levels of RelA or other NF-κB subunits were seen in the liver (Figure 1c; Supplementary Figure S1C), while no overt differences in the TNF response were observed in hepatocytes prepared from wild-type (WT) and RelA T505A mice when IκBα degradation, RelA Serine 536 phosphorylation and nuclear translocation were analysed (Figure 1d; Supplementary Figure S1D).

### The RelA T505A mouse reveals a role for RelA as a regulator of hepatocyte proliferation following partial hepatectomy

Following 70% partial hepatectomy in mice, the liver rapidly regenerates over a period of 5 days with peak cellular proliferation occurring after 36 h.^17^ In this model, a wave of highly synchronised hepatocyte cell-cycle entry occurs within hours as a consequence of the mechanical stress placed on the remnant liver, an increase in priming cytokines and growth factors, together with the requirement to maintain the mouse's metabolic demands. This process depends on NF-κB, although a recent report suggested that the RelA subunit was dispensable for liver regeneration.^22, 23^ However, subunit deletions can allow compensation by other NF-κB family members.^24^ By contrast, a knockin mutation that selectively removes one mechanism of regulation will behave differently if that pathway plays an important role in the process under study. Previous characterisation of RelA phosphorylation at Thr505 by Chk1 had implied a role in the cellular response to DNA replication stress.^12^ We were therefore interested in whether mutation of this site would affect proliferative responses *in vivo*. To address this, we investigated the response of WT and RelA T505A mice in a model of 70% partial hepatectomy.

**Figure 2:**
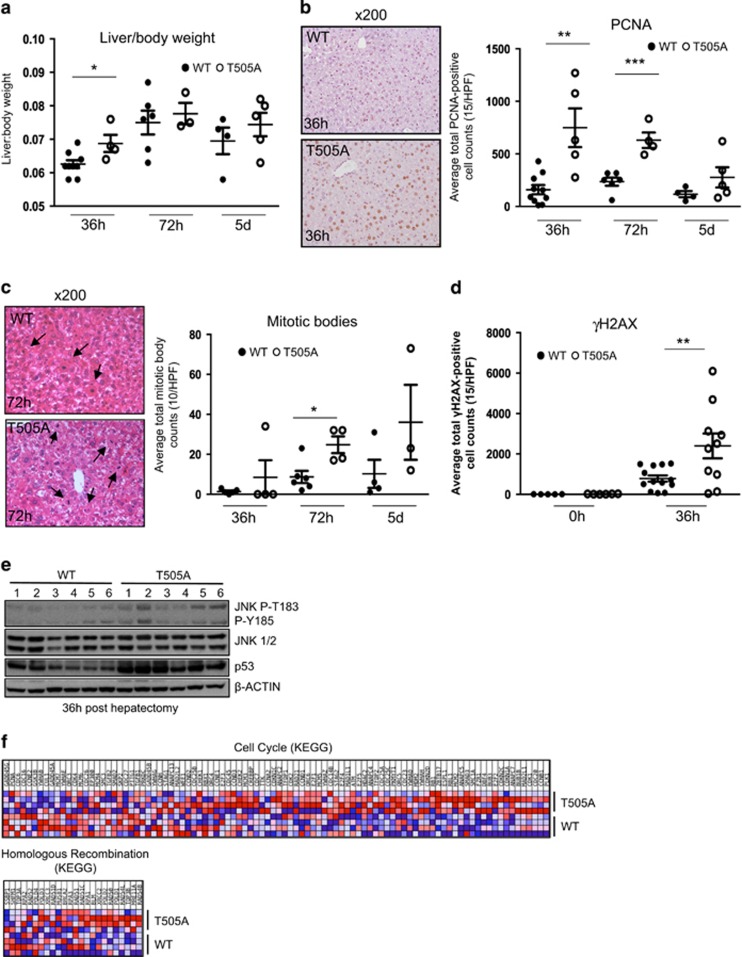
Increased hepatocyte proliferation in RelA T505A mice following partial hepatectomy. (**a**) Liver:body weight ratio at 36 h, 72 h and 5 days post partial hepatectomy (PhX) surgery in male WT and T505A mice. WT *n=*6; T505A *n=*6 per time point. (**b**–**d**) Mean number of cells/100 × field scoring positive for PCNA (**b**), mitotic bodies (**c**) and γH2AX (**d**) in WT and T505A liver tissue sections following PhX surgery. Representative images of each are shown. Photomicrographs are at × 200 magnification. *P<0.05, **P<0.01, ***P<0.001 (Unpaired Student's *t*-test). (**e**) Western blots with antibodies against total or phospho-JNK Thr183/Tyr185 and p53 in 36 h PhX liver samples from six WT and six T505A male mice. (**f**) GSEA of microarray data from livers of WT and RelA T505A mice 36 h following partial hepatectomy. Shown are the heat map results for KEGG-defined pathways for genes regulating cell cycle (KEGG_CELL_CYCLE, Enrichment Score −0.5287153; Normalised Enrichment Score −1.896711; Nominal *P*-value 0.0; false discovery rate *q*-value 0.004132211; familywise error rate *P*-value 0.014 and homologous recombination (KEGG _HOMOLOGOUS_RECOMBINATION; Enrichment Score −0.6658461; Normalised Enrichment Score −1.8532548; Nominal *P*-value 0.0; false discovery rate *q*-value 0.0063102846; Familywise error rate *P*-value 0.029). The heat map shows the clustered genes in the leading edge subsets of the gene set concerned (i.e., those showing the most differential expression between the data sets). The colours correspond to expression values, where red, pink, light blue and dark blue show the range of expression values from high to moderate to low to lowest, respectively.

Consistent with our hypothesis that mutation of RelA Thr505 would affect liver regeneration, male RelA T505A mice had a higher liver to body weight ratio relative to WT mice 36 h following hepatectomy (Figure 2a). Moreover, PCNA staining, mitotic body counts and BrdU incorporation analysis, all markers of cellular proliferation, showed higher levels in RelA T505A mice that extended to 72 h post hepatectomy (Figures 2b and c and Supplementary Figure S2A). This aberrant proliferation was associated with enhanced levels of γH2AX staining, a marker of DNA damage, together with raised levels of p53 protein and phospho-JNK (Figures 2d and e). 4-Hydroxy-2-nonenal staining revealed no significant increases (Supplementary Figure S2B), indicating that the increased replication stress and not enhanced levels of reactive oxygen species or liver peroxidation was the probable cause of this DNA damage response. These conclusions were supported by gene expression profiling of liver tissue samples before and 36 h after partial hepatectomy. Analysis of these data did not reveal a statistically significant gene signature associated with the T505A mutation (not shown), suggesting either that the effects seen result from the cumulative effects of relatively small increases in a large number of NF-κB target genes and are restricted to a subpopulation of cells, or that the effects of the Thr505 mutation occur at an earlier time point. However, gene set enrichment analysis (GSEA) revealed a significant upregulation of genes associated with proliferation and the cell cycle at 36 h post hepatectomy (Figure 2f and Supplementary Figure S2C). Moreover, consistent with the γH2AX analysis and raised p53 levels, significant induction of gene sets associated with DNA repair and replication was also observed (Figure 2f and Supplementary Figures S2C and D). Together these results demonstrate that RelA Thr505 phosphorylation provides an important regulatory mechanism to control the proliferative response and prevent cellular and genomic damage.

### RelA T505 regulates the response to acute and chronic liver injury

**Figure 3:**
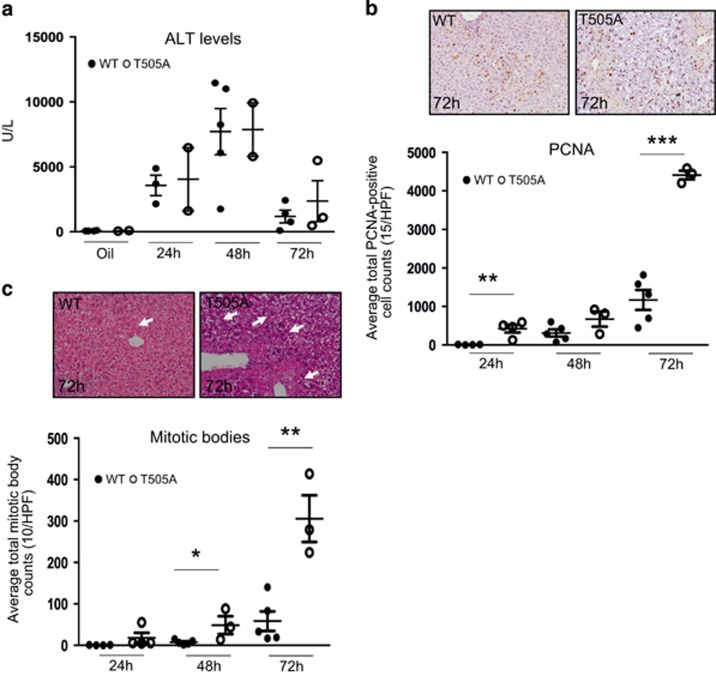
Increased hepatocyte proliferation in RelA T505A mice following acute CCl_4_ administration. (**a**–**c**) Serum transaminase, alanine aminotransferase measurements in arbitrary units/litre (U/L) (**a**), mean number of cells/field scoring positive for PCNA (**b**), and mitotic bodies (**c**) in WT and T505A liver tissue sections following acute CCl_4_ administration. Representative images of each are shown. Photomicrographs are at × 200 magnification. *P<0.05, **P<0.01, ***P<0.001 (Unpaired Student's *t*-test).

To confirm and extend this analysis, we investigated the response of mice to acute and chronic chemical liver injury with the potent hepatotoxin carbon tetrachloride (CCl_4_). CCl_4_ is metabolised primarily by the enzyme CYP2E1 in the liver to •CCl3, a trichloromethyl radical and a single IP dose of CCl_4_ will cause hepatocyte death, inflammation, activation of hepatic myofibroblasts and a compensatory asynchronous hepatocyte proliferation, which peaks after around 72 h. Following acute CCl_4_ administration, no significant differences in liver injury (as shown by elevation of serum transaminases), liver to body weight ratio, or hepatic myofibroblast activation (αSMA staining) was seen between WT and RelA T505A mice (Figure 3a and Supplementary Figures S3A and B). However, once again we observed a significant increase in markers of cell proliferation in RelA T505A mice (Figures 3b and c and Supplementary Figure S3C).

**Figure 4:**
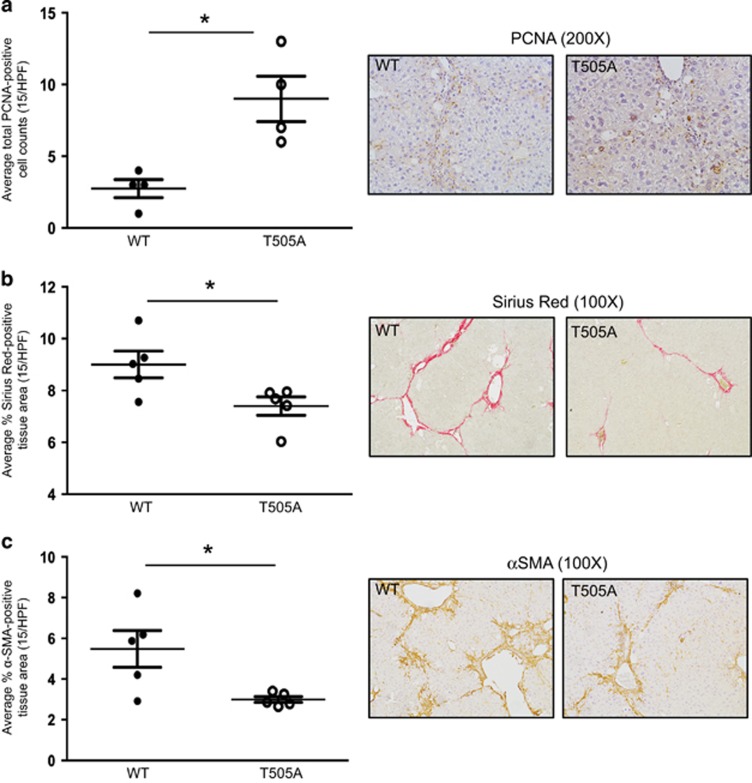
Increased hepatocyte proliferation and reduced fibrosis in RelA T505A mice following chronic CCl_4_ administration. (**a**–**c**) Mean number of cells/field scoring positive for PCNA (**a**), collagen (sirius red, SR) (**b**) and myofibroblasts (αSMA) (**c**) in WT and T505A liver tissue sections following 8 weeks of chronic CCl_4_ administration. Representative images of each are shown. Photomicrographs are at × 200 (PCNA) and × 100 (SR and αSMA) magnification. *P<0.05 (Unpaired Student's *t*-test).

Similar results were seen following chronic CCl_4_ administration. Here, after 8 weeks there was once again no change in liver body weight ratio, but significantly higher levels of markers of cell proliferation were again observed (Figure 4a and Supplementary Figure S4A). By contrast with the acute CCl_4_ model, chronic CCl_4_ administration resulted in a reduction in scar-forming hepatic myofibroblasts in RelA T505A mice, which was associated with a reduction in fibrosis, as assessed by sirius red staining for collagen (Figures 4b and c). Although reduced levels of liver injury were also seen in RelA T505A mice, these were not statistically significant (Supplementary Figure S4B).

### Earlier onset of liver cancer in RelA T505A mice

**Figure 5:**
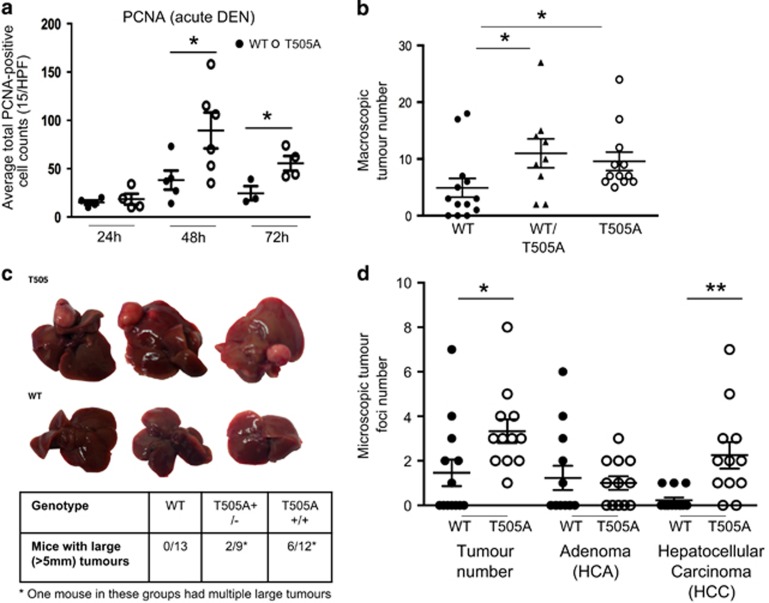
Early and increased onset of hepatocellular carcinoma in RelA T505A mice following DEN administration. (**a**) Mean number of cells/field scoring positive for PCNA in WT and T505A liver tissue sections following 24, 48 or 72 h of acute DEN administration. (**b**) Numbers of macroscopic tumours visible in WT and T505A livers 30 weeks after DEN administration. (**c**) Representative images of livers in WT and T505A livers 30 weeks after DEN administration. RelA T505A livers show large (>5 mm) tumours, summarised in the table shown. (**d**) Histological quantification of tumours into hepatocellular ademonas and carcinoma in liver sections from DEN-treated WT and T505A mice. An expert pathologist scored all pathology. *P<0.05, **P<0.01 (Unpaired Student's *t*-test).

Previous *in vitro* studies of RelA Thr505 phosphorylation had all pointed to this modification providing a mechanism to suppress the tumour-promoting functions of RelA.^11, 12, 15, 16^ The data from partial hepatectomy and liver injury models above, where consistently higher levels of cell proliferation, accompanied by higher levels of DNA damage were seen in RelA T505A mice, were all consistent with this hypothesis. We therefore next investigated if RelA T505A mice would exhibit earlier onset of tumorigenesis in the *N*-nitrosodiethylamine (DEN) model of hepatocellular carcinoma.^25^ DEN is a DNA alkylating agent and so we first investigated whether acute administration, which promotes hepatocyte death followed by compensatory proliferation, resulted in any observable differences between WT and RelA T505A mice. Consistent with earlier data, an enhanced proliferative response was observed in RelA T505A mice, with no differences in liver/body weight ratio and only a small difference in liver damage being seen after 72 h (Figure 5a and Supplementary Figures S5A and C). This result suggested that RelA T505A mice could display differences in tumour development following DEN treatment. DEN was, therefore, administered to 15-day-old WT and RelA T505A mice and tumour growth was evaluated after 30 weeks. At this point tumours start to become visible on the liver surface in WT mice and therefore this is the optimal time to investigate if the RelA T505A mutation resulted in earlier onset of tumorigenesis. Analysis of these tumours revealed that both homozygous and heterozygous RelA T505A mice had significantly higher numbers of visible liver tumours (Figure 5b). Moreover, RelA T505A mice were more likely to display large (>5 mm diameter) tumours (6/12 for homozygotes, 2/9 for heterozygotes) compared to WT mice, where none were seen at this time point (0/13) (Figure 5c).

More detailed histological analysis of liver tissue sections by an expert pathologist confirmed the visible differences in tumour numbers between RelA T505A mice and WT mice (Figure 5d and Supplementary Table T1). Interestingly, while the numbers of adenomas are broadly equivalent between the two mice strains (16 foci from 13 WT mice, 12 foci from 12 RelA T505A mice), the hepatocellular carcinoma numbers were dramatically different. Here the 12 RelA T505A mice had 27 hepatocellular carcinoma versus only three seen in the WT mice (Figure 5d; Supplementary Figure S5D and Supplementary Table T1). Although NF-κB can promote the development of inflammation-associated cancers,^2, 18, 21^ no differences in the levels of inflammation or fibrosis were observed between WT and RelA T505A mice (Supplementary Table T1). Consistent with this, when we examined cytokine serum levels in mice from the DEN model by multiplex ELISA, the only one to show a significant difference in the RelA T505A mice was KC/GRO (CXCL1), a cytokine primarily expressed by macrophages, where levels were significantly raised (Supplementary Figure S6). Q-PCR analysis of tumour tissue from DEN-treated livers did not show a statistically significant increase in KC/GRO mRNA levels in RelA T505A mice, although there was a trend towards higher levels (Supplementary Figure S6J). Although interleukin-6 levels were raised in a few RelA T505A mice, overall the results were not significant. No changes were seen with TNFα, interleukin-1β and other cytokines examined. Therefore, together with there being no indication of changes in inflammation associated with the DEN tumours in RelA T505A mice, we conclude that the effects we see on tumour growth do not primarily arise through an inflammatory mechanism, although we cannot rule out a potential role for KC/GRO.

Although broadly similar to WT mice, the background liver of RelA T505A NF-κB knockin mice did show less severe DEN-induced fibrotic injury (stage 2 fibrosis in all cases versus mixed stage 2 (6/13) to stage 3 (7/13) in WT mice). There was also increased steatosis in RelA T505A mice (mild to severe in 10/12 cases of macrovesicular or mixed type versus mild to moderate macrovesicular or mixed steatosis without steatohepatitis in 7/13 cases of WT mice) (Supplementary Table T1).

**Figure 6:**
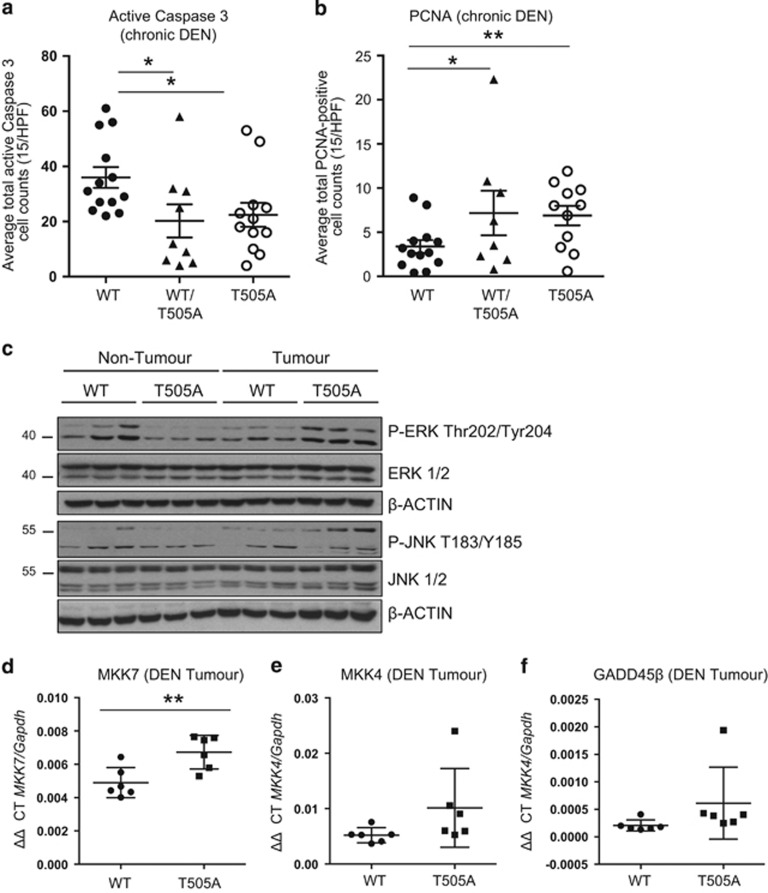
Altered apoptosis and ERK/JNK signalling in DEN-treated livers from RelA T505A mice. (**a**, **b**) Mean number of cells/field scoring positive for active Caspase 3 (**a**) and PCNA (**b**) in WT and T505A livers 30 weeks after DEN administration. (**c**) Western blots with antibodies against total or phospho-Thr183/Tyr185 JNK, total or phospho-Thr202/Tyr204 ERK and total or phospho-Thr180/Tyr182 p38 kinases in tumour and non-tumour tissue from three different WT and T505A livers 30 weeks after DEN administration. (**d**–**f**) RT-qPCR data showing relative MKK7 (**c**), MKK4 (**d**) and Gadd45β (**b**) mRNA expression tumour tissue from WT and T505A livers 30 weeks after DEN administration. Data represent mean±s.e.m.; each point is an individual mouse. *P<0.05, **P<0.01 (Unpaired Student's *t*-test).

Further analysis of these tumours revealed overall lower levels of apoptosis, but increased levels of proliferation in DEN-treated livers from RelA T505A mice (Figures 6a and b; Supplementary Figures S7A and B). Furthermore, interesting differences in the activity of cell signalling pathways was observed between tumour and non-tumour tissue. Here, reduced levels of phospho-ERK and phospho-JNK signalling were seen in extracts from non-tumour tissue, while the opposite was found in tumour samples (Figure 6c). JNK activity in the liver has been linked to cancer, fibrosis and steatosis.^17, 26^ Consistent with this raised level of JNK activity in tumours, a modest but significant increase in the mRNA levels of MKK7 (mitogen-activated protein kinase kinase 7), a kinase known to activate JNK signalling in the liver, was seen (Figure 6d).^17, 27^ No significant effect was seen with the related kinase MKK4, although some RelA T505A tumour samples did display higher levels (Figure 6e). By contrast, although NF-κB-dependent control of JNK activity in the liver has been linked to regulation of its target gene Gadd45β,^17^ we did not observe any changes in its expression in RelA T505A tumours (Figure 6f).

Taken together, these results demonstrate that the T505A mutation results in a more tumour-promoting form of RelA that leads to the earlier onset of more aggressive hepatocellular carcinoma.

## Discussion

**Figure 7:**
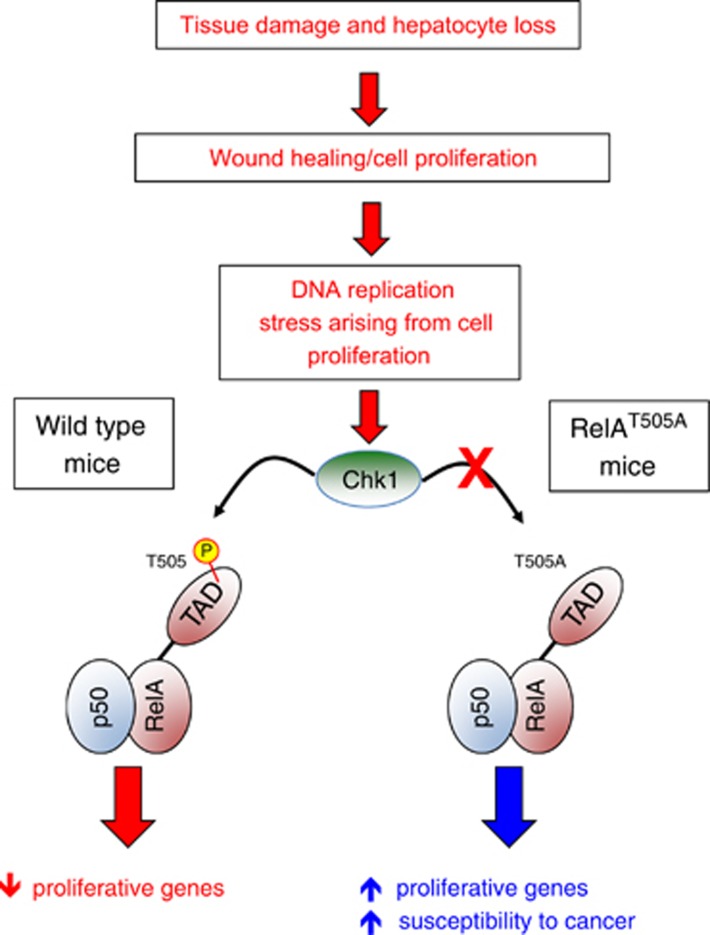
Model showing the effects of the RelA T505A mutation. In WT mice, tissue damage and hepatocyte loss leads to compensatory wound healing and cell proliferation. We propose that, as a consequence of this, DNA replication stress will lead to checkpoint kinase activation and Chk1-mediated phosphorylation of RelA at Thr505 (although other kinases could also perform this role). This in turn results in suppression/modulation of RelA transcriptional activity. However, in RelA T505A mice this pathway is defective, meaning that RelA is not subject to checkpoint kinase regulation, leading to an enhanced proliferative response and other effects, resulting in earlier onset of hepatocellular carcinoma.

Here we have demonstrated that RelA Thr505 phosphorylation is an important physiological regulator of NF-κB function in the liver. Modification of this site provides a mechanism to antagonise and limit aspects of RelA function associated with its tumour-promoting activities (Figure 7). Although PTMs of RelA have been highly studied, little is known about their physiological roles. Moreover, most attention has been focused on a relatively small subset of these modifications, whereas the range of PTM types and number of sites capable of being modified is very high.^4, 7^ This diversity provides a mechanism to control the functional consequences of NF-κB activation by allowing selective regulation of NF-κB gene targets.

*In vitro* analysis of these modifications is hampered by the problems associated with subunit overexpression or non-physiological responses associated with the analysis in cancer cell lines. Therefore, to learn more about the significance of RelA Thr505 phosphorylation, we created a knockin mutant mouse. Study of the RelA subunit in adult animals has been hampered by the lethality of the gene knockout resulting from TNF-induced liver apoptosis.^9^ Although an increasing number of studies have employed a conditional knockout of RelA (for example Ringelhan *et al.*,^23^ *Algul et al.*,^28^ Stein *et al.*^29^) a consequence of this is that much is still not known about RelA function in mature mice, with many assumptions being based on the effect of IKK mutants that are now known to have a variety of NF-κB independent functions.^30^ Moreover, gene knockouts inactivate a wide range of functions that can potentially mask other, subtler but still important effects. For example, the RelA knockout has no effect in the partial hepatectomy model, while our T505A knockin does^22, 23^ (Figure 2). To date, the only other published analysis of RelA phospho-site knockin mutant mice involves analysis of the Ser276 residue. The effect of mutating this site to alanine resulted in a form of RelA that functions as a transcriptional repressor.^31^ This resulted in gene repression leading to embryonic death at different stages due to variegated developmental abnormalities.^31^ However, this site is functionally distinct from the Thr505 residue, with this latter modification not being required for nuclear translocation or gene activation in response to inflammatory stimuli.^11, 12, 16^ Rather, it provides a mechanism through which certain types of DNA damage, such as replication stress, antagonise NF-κB activity. However, *in vivo* evidence for the importance of this pathway was lacking. Here, we have established the functional importance of this pathway *in vivo.* We demonstrate that in the liver the response to injury has an important modulatory effect on NF-κB, suppressing the pro-proliferative functions associated with NF-κB's tumour-promoting abilities (Figure 7). We propose that further knockin mutagenesis of RelA and other NF-κB subunits will provide a clearer route to define its role *in vivo* and reveal unknown functions not seen in previous mouse models.

In addition to an increase in proliferation, other effects were also observed, such as a reduction in fibrosis in RelA T505A mice with the chronic CCl_4_ model (Figure 4). This effect is likely to be a consequence of reduced numbers of scar-forming cells, but we do not exclude the possibility that there could be an inflammatory contribution to the phenotype. An increase in steatosis was also seen in the livers of RelA T505A mice in the DEN model of hepatocellular carcinoma. Steatosis has been linked with increased susceptibility to liver cancer, even in the absence of fibrosis.^32^ This hepatic lipid accumulation can induce metabolic changes, increase reactive oxygen species production and induce signalling pathways including JNK.^26^ The combination of steatosis and increased hepatocyte proliferation may cooperate to accelerate tumour development in the RelA T505A NF-κB knockin mice.

Given the effect of the RelA T505A mutation, it might be expected that this site or region of the RelA transactivation domain would be also mutated in human cancer or that SNPs in this region would predispose to liver or other diseases. However, analysis of the literature or the Catalogue of Somatic Mutations in Cancer (http://cancer.sanger.ac.uk/cosmic) database demonstrates very few alterations in the motif containing RelA Thr505 (not shown). But alterations in the coding sequence of RelA and other NF-κB subunits, despite their involvement in many diseases, are very rare.^4^ This may be a reflection of the NF-κB response having many different functional elements and so subunit mutations would have the effect of enhancing certain aspects of NF-κB behaviour at the expense of others. Consequently, if a tumour requires the overall NF-κB response to promote its growth and survival, such mutations would not be selected. Therefore, while in the context of an experimental system, such as those used here, we see effects that enhance tumorigenesis, in reality there may be negative consequences, that mean such mutations have an overall detrimental effect on the tumour.^4^

We have not yet been able to demonstrate phosphorylation of the Thr505 in liver tissue. This may reflect the relatively poor quality of phospho-specific antibodies to this site. Alternatively, phosphorylation of this site may either be very labile and lost during the process of making protein extracts from the liver, be restricted to a limited to a subpopulation of cells or be specific to a cell cycle stage.

An important question for future studies will be whether this mutation affects a specific and common set of target genes in all cell types or whether any effects on gene expression are context dependent. Moreover, as our analysis of gene expression following partial hepatectomy did not reveal gene-specific effects, it is possible that this site exerts a modulatory effect on multiple NF-κB targets, the cumulative effect of which leads to the changes in cell behaviour. Alternatively, these effects might be restricted to a subset of liver cells or a specific time point in the experimental procedure. Nonetheless, we have established for the first time *in vivo* the importance of the RelA Thr505 motif for both chemical and surgically induced liver regeneration together with carcinogen-induced hepatocellular carcinoma.

## Materials and methods

### Mice

All experiments were approved by Newcastle University's Animal Welfare and Ethical Review Board. Animals were bred in the Newcastle University animal unit, maintained as specific pathogen free according to the FELASA Guidelines. Work was carried out under project and personal licences approved by the Home Office. Transgenic RelA T505A mice were generated by Taconic Artemis (Cologne, Germany) using C57Bl/6 ES cells. All mice were maintained on a pure C57BL/6 background. No blinding of groups in mouse studies was performed. All mice were designated to an experimental group dependent on their genotype.

### Genotyping of *Rel*^T505A/T505A^ mice

Genotyping was performed by polymerase chain reaction using genomic DNA isolated from ear clips. The WT or *Rel*^T505A/T505A^ alleles were amplified using Eurogentec forward 5′-AGCCATGCTCCTGTCAAACC-3′ and reverse 5′-CAGGGATTATTTAGTCCCTTGG-3′ specific primers for 35 cycles; denaturation: 95 °C, 30 s; annealing: 56 °C, 40 s; extension: 72 °C, 1 min). Amplification of a single product of 241 or 360 bp corresponds to WT or T505A^+/+^ allele, respectively.

### Murine hepatocyte isolation

Mouse primary hepatocytes were isolated from aged-matched 8–12 weeks WT and RelA T505A males as described.^33^ Hepatocytes were washed and media was changed to 0% FBS 3–4 h after isolation. TNF-α 50 ng/ml (Peprotech, London, UK) treatment was performed ~20 h post isolation for 10 or 30 min.

### Immunofluorescence

WT and RelA T505A hepatocytes were cultured in collagen I-coated glass coverslips, fixed with formalin and permeabilised with 0.1% saponine/0.5% BSA solution. After blocking with 3% BSA, RelA antibody was added for 120 min. After washing, secondary donkey anti-rabbit Alexa 594 (Invitrogen, Paisley, UK) was incubated for 60 min. Coverslips were mounted with ProLong Diamond DAPi-conjugated mounting medium (A-21207, Invitrogen). Pictures of slices with 0.5 *μ*m thickness were taken using a Multiphoton Nikon (Kingston upon Thames, UK) A1 confocal microscope at × 600 oil. An electronic zoom of magnification 1.8 was made of a region of interest. Image analysis was performed using Image J software (NIH, Bethesda, MA, USA).

### Partial hepatectomy

Seventy percent liver partial hepatectomy (PhX) was performed on 10–12-week-old male littermates as previously described.^34^ At 36 h, 72 h and 5 days following PhX, the mice were humanely killed and liver tissue was harvested. Appropriate pain relief was given.

### *In-vivo* models of liver injury

For acute CCl_4_ treatment, WT and T505A 8–10-week-old male littermates were injected intraperitoneally (IP) with CCl_4_ at a dose of 2 μl (CCl_4_:olive oil, 1:1 [v:v])/g body weight). Acute DEN, 8-week-old mice were given 80 mg/kg intraperitoneal injection to induce liver DNA damage. Mice were humanely killed, then liver and serum were harvested at 24, 48 and 72 h. For chronic CCl_4_ treatment, 8–12 week old male wt and T505A male littermate mice were injected with CCl_4_ intraperitoneally (IP) twice a week at a dose of 2 μl (CCl_4_:olive oil, 1:3, [v:v])/g body weight during 8 weeks. Mice were humanely killed, then liver and serum was harvested. Serum transaminases were measured in the Clinical Biochemistry department at the Royal Victoria Infirmary, Newcastle.

### *In-vivo* models of liver cancer

Day 15 mice were given 30 mg/kg DEN in 0.9% saline by IP injection to induce liver cancer. Mice were humanely killed, and liver and serum were harvested at 30 weeks post DEN. Serum transaminases were measured in the Clinical Biochemistry department at the Royal Victoria Infirmary, Newcastle.

### Immunohistochemistry

Formalin-fixed paraffin-embedded liver sections were dewaxed and hydrated. Endogenous peroxidase activity was blocked with hydrogen peroxide and antigen retrieval was achieved using 1 mM EDTA for γH2A.X, 10 mM sodium citrate buffer, pH 6.0 for active caspase 3 and BrdU and 0.0005% Trypsin for 20 min at 37 °C and PCNA. Tissue was blocked using an Avidin/Biotin Blocking Kit (Vector Laboratories, Peterborough, UK) followed by 20% swine serum in PBS and then incubated with primary antibodies overnight at 4 °C. The next day slides were washed and incubated with biotinylated swine anti-rabbit followed by Vectastain Elite ABC Reagent (Peterborough, UK). Antigens were visualised using DAB peroxidase substrate kit and counterstained with Mayer's hematoxylin. Immuno-stained cells were manually counted and expressed as the mean number of positive cells per field in 15 high-power fields at × 20 magnification or 10 fields at × 10 magnification. For all histological studies, the stained slides were blinded (coded) prior to analysis.

#### H&E and Sirius Red

Formalin-fixed and paraffin-embedded sections were dewaxed, hydrated and then stained with either H&E or 0.1% Sirius Red Picric solution following standard procedures.

### Multi-SPOT Assay System (MSD) V-PlexTM serum analysis

Serum for 10 × WT and 10 × RelA T505A 30-week-old-DEN-injected animals were analysed for cytokine levels in triplicate using the MSD V-Plex assay system. Samples were diluted 1:2 and incubated overnight. The assay was performed according to the manufacturer's instructions.

### Cell extracts for western blotting

Mouse liver tissue was homogenised in Phosphosafe extraction buffer (Millipore, Watford, UK) following manufacturer's protocols in Precellys 24 ceramic mix bead tubes (Stretton Scientific Ltd, Stretton, UK) in a Precellys 24 homogeniser (Stretton Scientific Ltd) at 6500 r.p.m. for 30 s. BCA (Thermo Scientific Ltd, Loughborough, UK) analysis was performed to normalise protein concentration.

### Quantitative PCR

Quantitative PCR was performed as described previously.^35^ Qiagen Quantitect primer assays were used for RT-qPCR analysis of genes of interest. Housekeeping control Gapdh primers were from Eurogentec (forward 5′-GCTACACTGAGGACCAGGTTG-3′ and reverse 5′-GCCCCTCCTGTTATTATGGGG-3′).

### Microarray analysis

Total RNA was extracted from snap-frozen livers samples from control and 36-h PhX livers. Briefly, frozen samples were homogenised using Precellys 24 ceramic mix bead tubes (Stretton Scientific Ltd) in a Precellys 24 homogeniser (Stretton Scientific Ltd) at 6500 rpm for 30 s according to the Qiagen RNeasy mini kit instruction manual. After, samples were passed through Qiashredders (Qiagen, Crawley, UK) and RNA was purified following instructions from the Qiagen RNeasy mini kit. Microarray analysis was performed by Cambridge Genomic Services.

### Bioinformatics analysis

The Illumina mouse WG-6 Expression BeadChip data were background corrected in Illumina GenomeStudio, subsequent analysis proceeded using the lumi and RankProd packages in R (Bioconductor, Seatlle, WA, USA).^36, 37, 38^ Variant Stabilisation Transform and Robust Spline Normalisation were applied in lumi. Differential expression was detected using Rank Products analysis. A list of genes for each comparison was generated using 0.05 percentage of false positives as a cutoff. Expression profiles of the 18 106 probes that passed a detection *P*-value filter on all of the microarrays were used for GSEA.^39^ GSEA is used to determine the differences between an experimental gene set and a selected gene list from the Molecular Signatures Database (MSigDB, Broad Institute, Cambridge, MA, USA), an annotated database of gene sets. GSEA can be downloaded from http://www.broadinstitute.org/gsea/downloads.jsp.

### Other statistical analyses

All studies were performed on at least three independent triplicates. GraphPad Prism version 5 software was used to calculate unpaired Student's *t*-tests and results are reported as mean±s.e.m. *P* values<0.05 were considered significant.

### Antibodies

Anti-anti-p53 (#2524), JNK (#9252), phospho-SAPK/JNK (Thr183/Tyr185) (81E11) (#4668), ERK (#9102), Phospho-p44/42 MAPK (Erk1/2) (Thr202/Tyr204) (#4370), p100/p52 (#4882), RELB, phospho-S536 RELA (#3033), IκB-α (#9242), active caspase 3 (#9664) and γH2A.X (#9718) were from Cell Signaling Technology (Hitchin, UK). Anti-RELA (sc372) and c-Rel (sc71) were from Santa Cruz (Wembley, UK). Anti-β-ACTIN (A5441) was from Sigma (Gillingham, UK). Anti-PCNA (ab2426), and p105/p50 (ab32360) were from Abcam (Cambridge, UK). Anti-BrdU (#347580) was from BD (Oxford, UK). Anti-rabbit IgG (Sigma A6154 and Cell Signaling #7074) and anti-mouse IgG (Sigma A9044) HRP-linked secondary antibodies were used for western blot detection.

### Accession numbers

Microarray data have been submitted to ArrayExpress with accession number: E-MTAB-3596.
