# Supplementary material for: A RelA(p65) Thr505 phospho-site mutation reveals an important mechanism regulating NF-κB-dependent liver regeneration and cancer
Source: Oncogene. 2016 Feb 8;35(35):4623–32. doi: 10.1038/onc.2015.526 (PMC4862573; doi:10.1038/onc.2015.526)
Supplement: Supplementary Figures [file onc2015526x1.pdf]

Figure S1

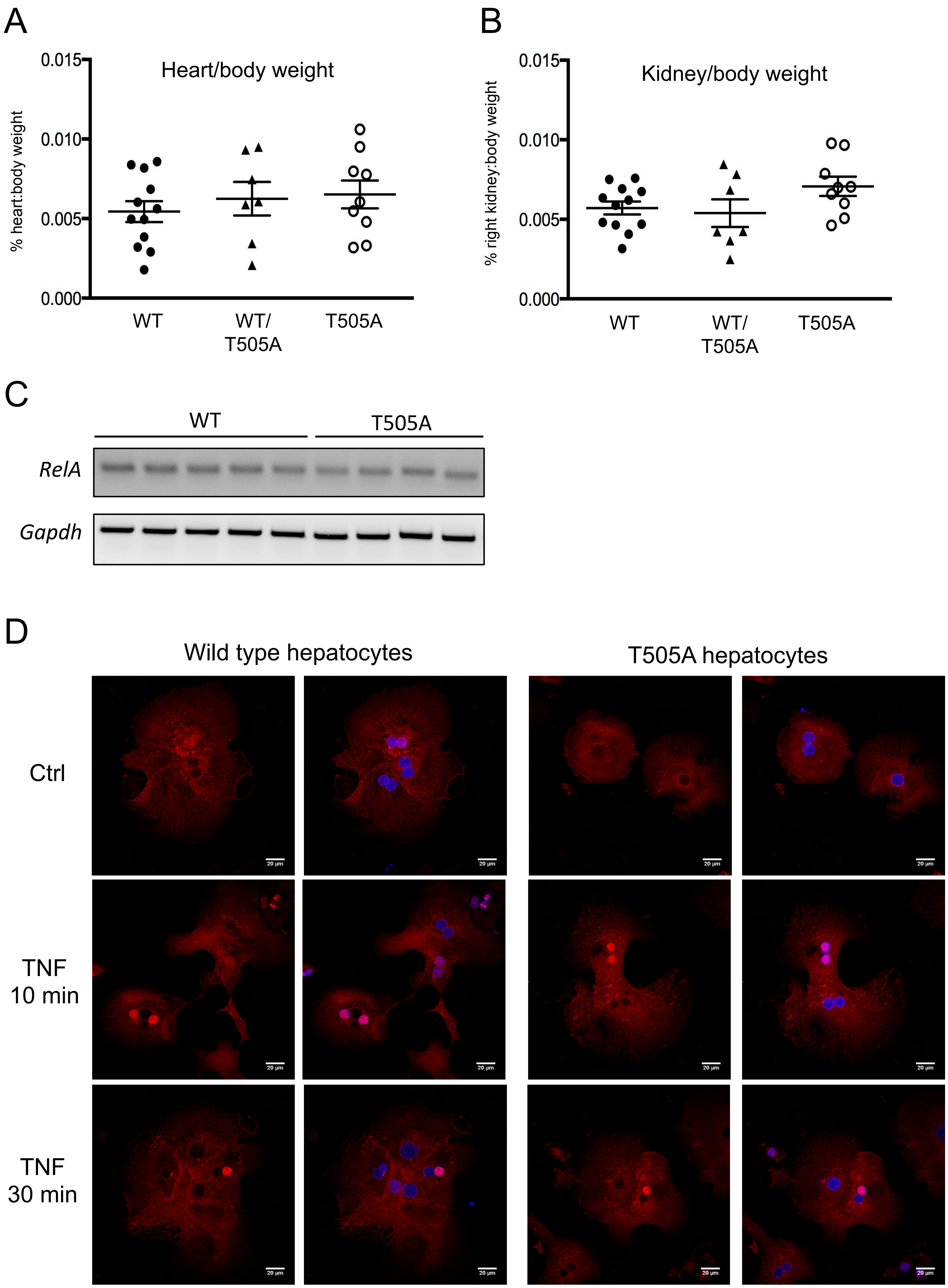
A
Heart/body weight
% heart:body weight
0.015
0.010
0.005
0.000
WT
WT/
T505A
T505A
B
Kidney/body weight
% right kidney:body weight
0.015
0.010
0.005
0.000
WT
WT/
T505A
T505A
C
WT
T505A
RelA
Gapdh
D
Wild type hepatocytes
T505A hepatocytes
Ctrl
TNF
10 min
TNF
30 min

Figure S2

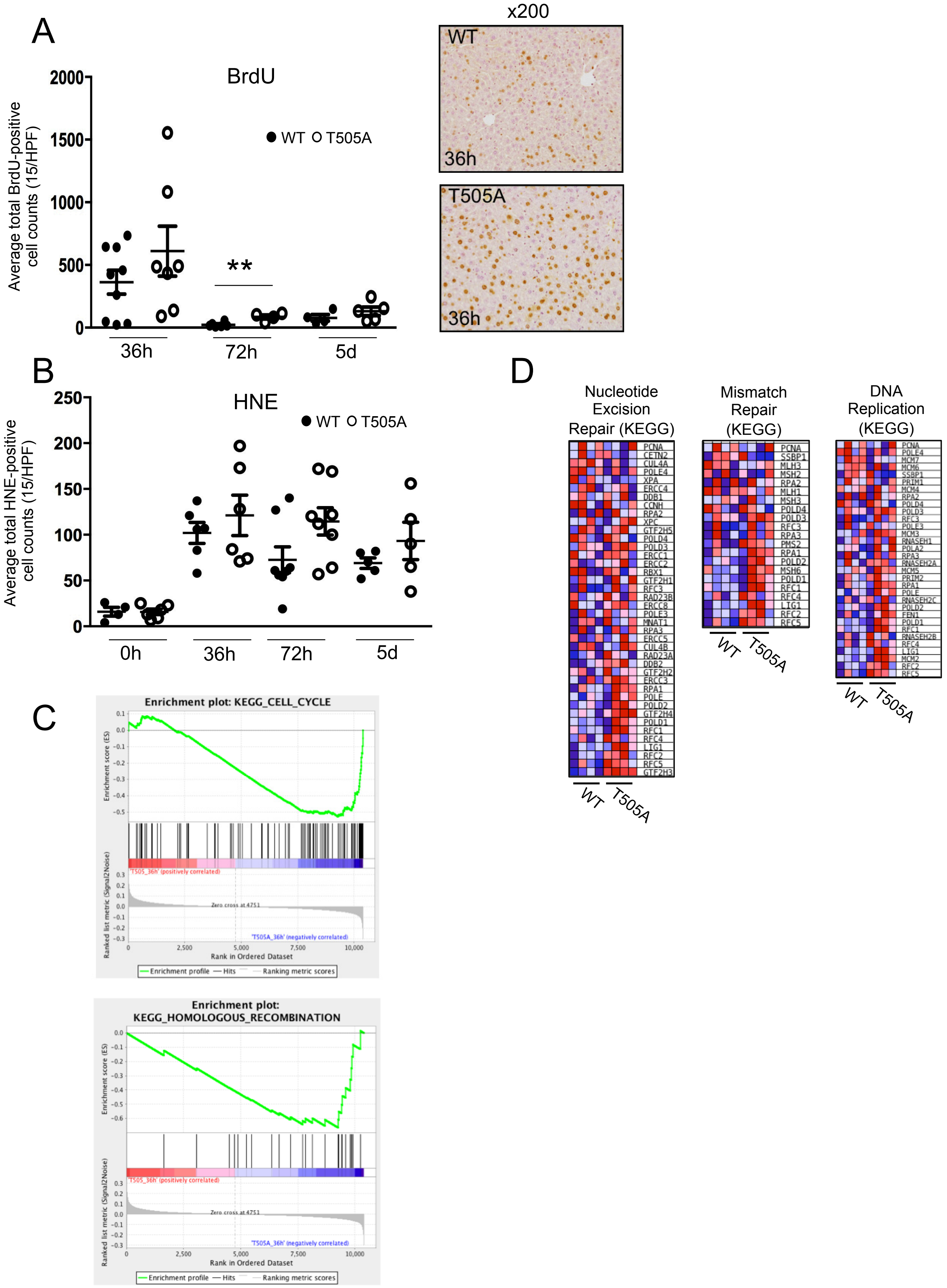
A
x200
BrdU
Average total BrdU-positive cell counts (15/HPF)
2000
1500
1000
500
0
● WT ○ T505A
**
36h
72h
5d
WT
36h
T505A
36h
B
HNE
Average total HNE-positive cell counts (15/HPF)
250
200
150
100
50
0
● WT ○ T505A
0h
36h
72h
5d
C
Enrichment plot: KEGG_CELL_CYCLE
Enrichment score (ES)
Ranked list metric (Signal2Noise)
'T505_36h' (positively correlated)
Zero cross at 4751
'T505A_36h' (negatively correlated)
Rank in Ordered Dataset
Enrichment profile
Hits
Ranking metric scores
Enrichment plot: KEGG_HOMOLOGOUS_RECOMBINATION
Enrichment score (ES)
Ranked list metric (Signal2Noise)
'T505_36h' (positively correlated)
Zero cross at 4751
'T505A_36h' (negatively correlated)
Rank in Ordered Dataset
Enrichment profile
Hits
Ranking metric scores
D
Nucleotide Excision Repair (KEGG)
Mismatch Repair (KEGG)
DNA Replication (KEGG)
WT
T505A
WT
T505A
WT
T505A

Figure S3

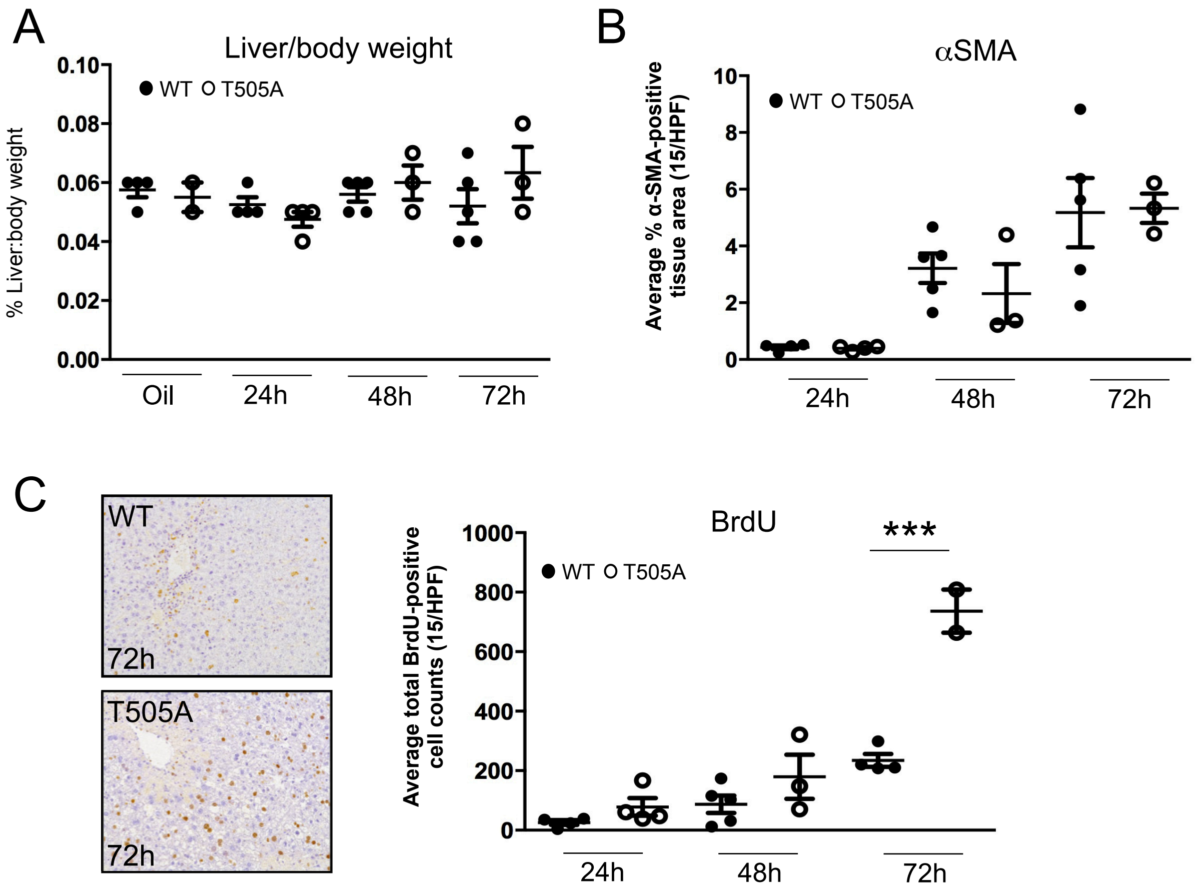
A
Liver/body weight
WT T505A
% Liver:body weight
0.10
0.08
0.06
0.04
0.02
0.00
Oil
24h
48h
72h
B
αSMA
WT T505A
Average % α-SMA-positive tissue area (15/HPF)
10
8
6
4
2
0
24h
48h
72h
C
WT
72h
T505A
72h
BrdU
***
WT T505A
Average total BrdU-positive cell counts (15/HPF)
1000
800
600
400
200
0
24h
48h
72h

Figure S4

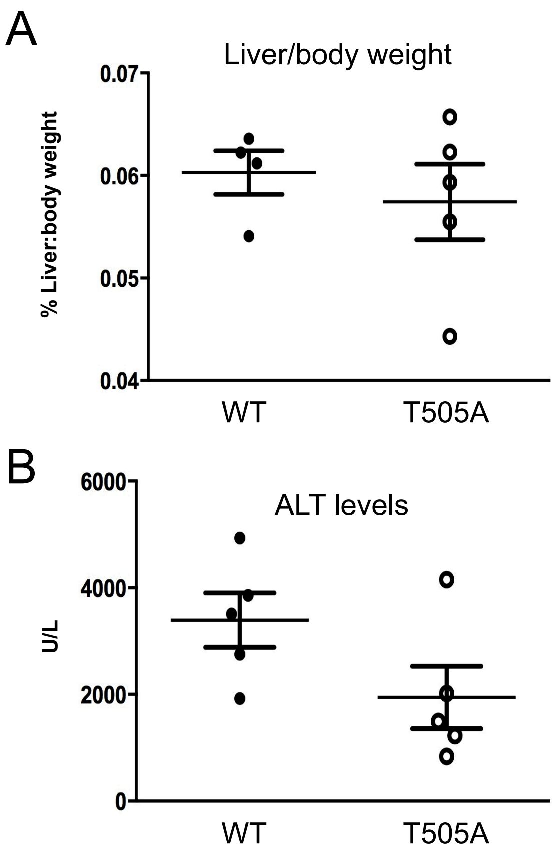
A
Liver/body weight
% Liver:body weight
0.07
0.06
0.05
0.04
WT
T505A
B
ALT levels
U/L
6000
4000
2000
0
WT
T505A

Figure S5

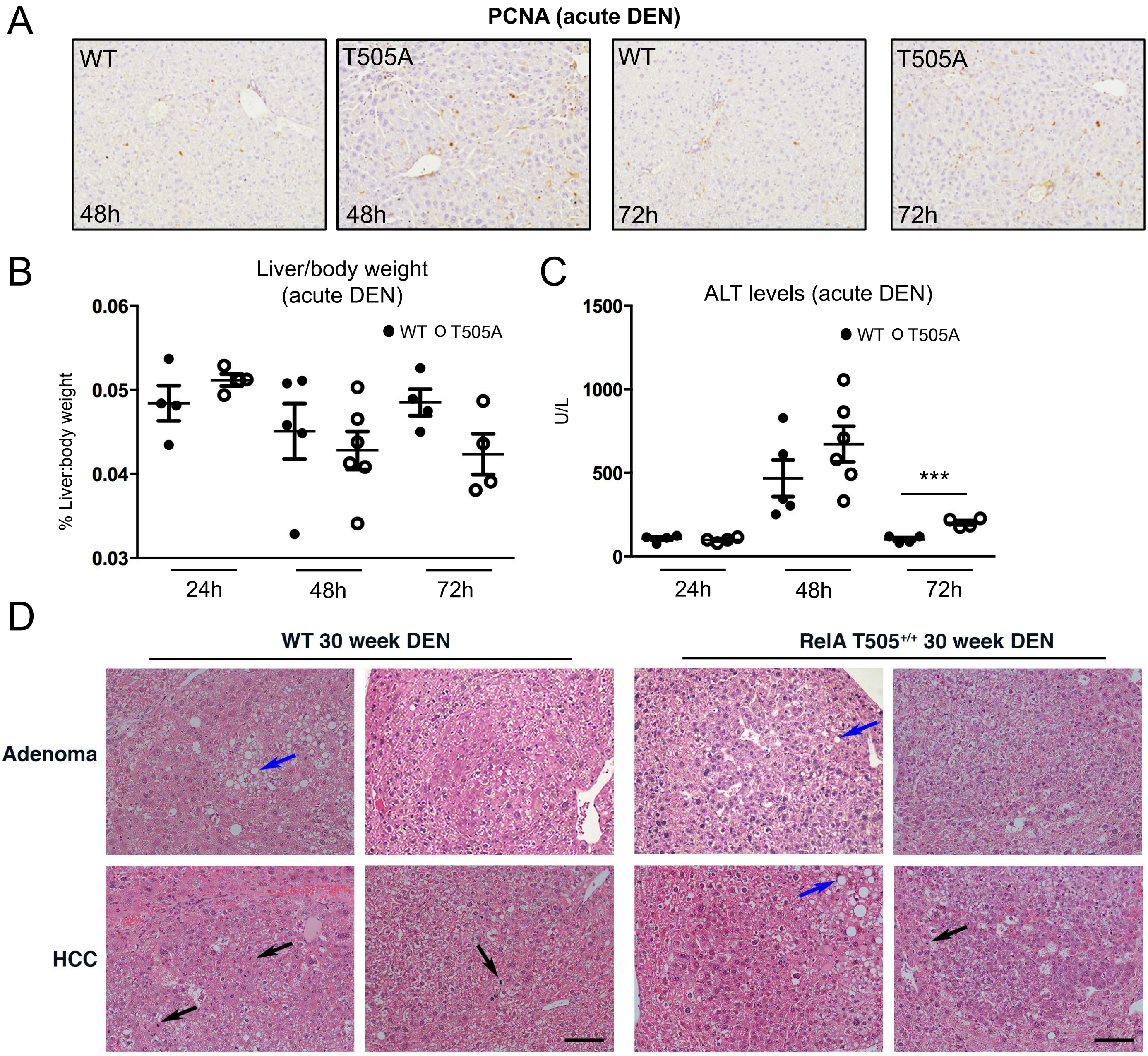

A
PCNA (acute DEN)
WT
48h
T505A
48h
WT
72h
T505A
72h
B
Liver/body weight (acute DEN)
● WT ○ T505A
% Liver:body weight
0.06
0.05
0.04
0.03
24h
48h
72h
C
ALT levels (acute DEN)
● WT ○ T505A
U/L
1500
1000
500
0
***
24h
48h
72h
D
WT 30 week DEN
RelA T505+/+ 30 week DEN
Adenoma
HCC

Figure S6

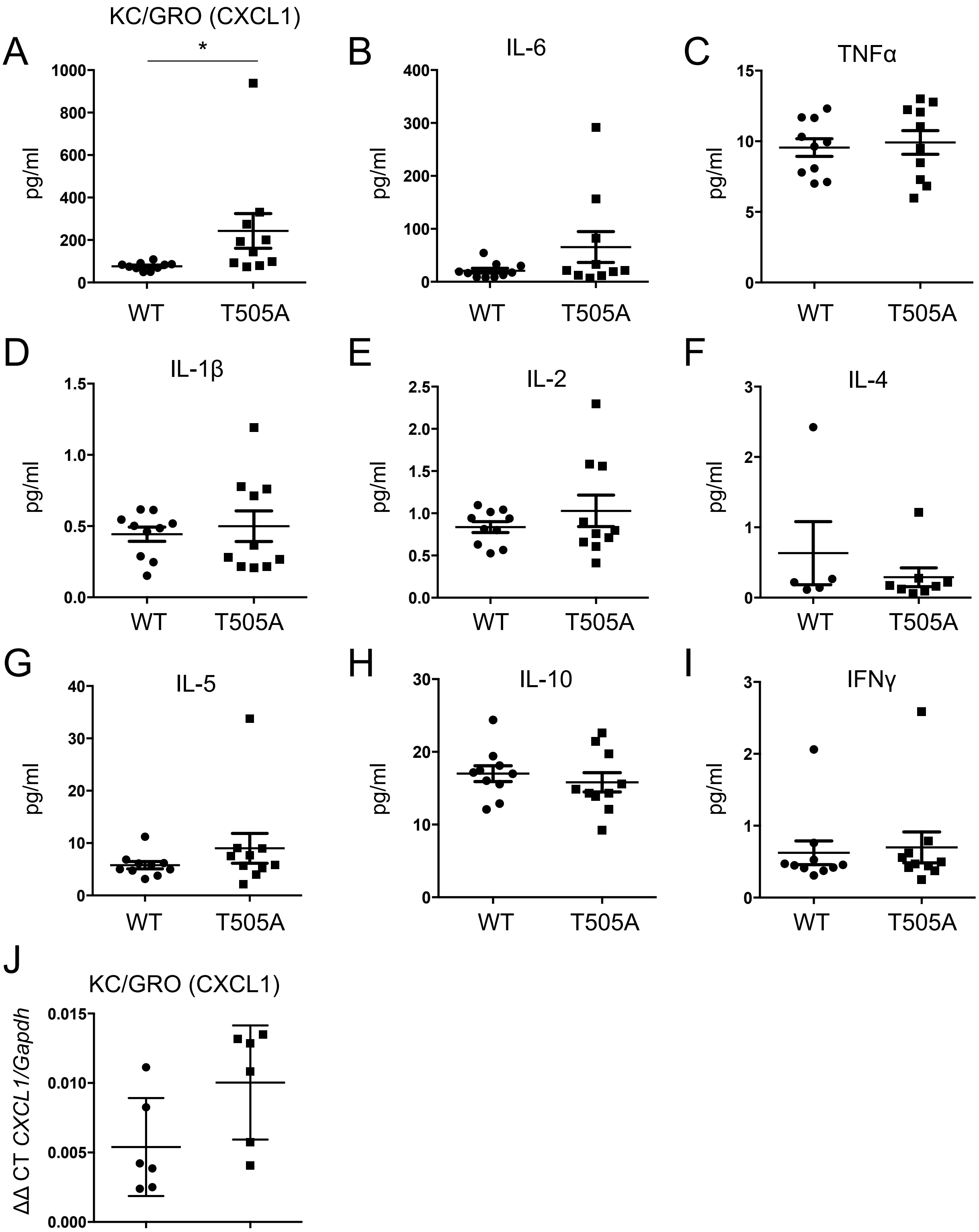
A
KC/GRO (CXCL1)
*
B
IL-6
C
TNFα
D
IL-1β
E
IL-2
F
IL-4
G
IL-5
H
IL-10
I
IFNγ
J
KC/GRO (CXCL1)
pg/ml
WT
T505A
ΔΔ CT CXCL1/Gapdh

Figure S7

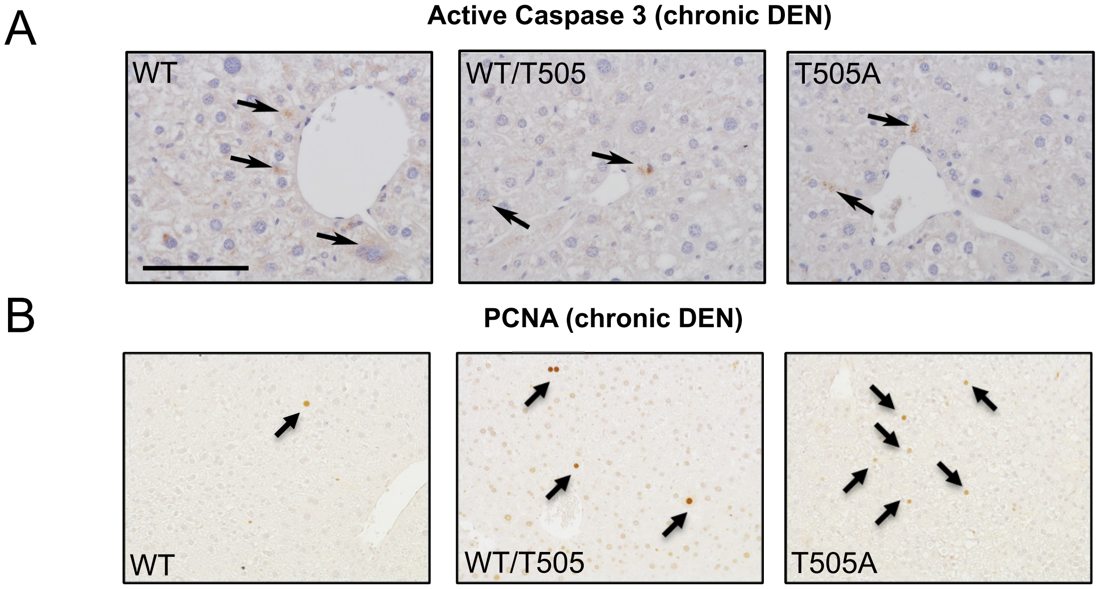
A
Active Caspase 3 (chronic DEN)
WT
WT/T505
T505A
B
PCNA (chronic DEN)
WT
WT/T505
T505A

Table T1

Genotype	Adenoma (HCA)	HCC	Well differentiated HCC	Total tumor number	Regenerative nodule/foci	Steatosis	Inflammation	Fibrosis	Mitotic bodies
WT 1	0	0	NO	0	0	MILD	Rare	MILD	NO
WT 2	0	0	NO	0	0	NO	MILD	YES	YES
WT 3	0	0	NO	0	2	NO	MILD	YES	NO
WT 4	1	1	NO	2	1	NO	MILD	YES	NO
WT 5	4	0	NO	4	0	MILD	MILD	YES	YES
WT 6	6	1	NO	7	0	MILD	MILD	YES	NO
WT 7	0	0	NO	0	0	MILD	Rare	MILD	NO
WT 8	0	0	NO	0	0	NO	MILD	YES	NO
WT 9	0	0	NO	0	0	MODERATE	MILD	YES	YES
WT 10	0	0	NO	0	0	NO	MILD	YES	NO
WT 11	3	0	NO	3	3	NO	MILD	MILD	YES
WT 12	2	0	NO	2	0	MIXED	MILD	YES	NO
WT 13	0	1	Yes (1)	1	0	MODERATE	MILD	MILD	YES
RelA T505A 1	2	1	Yes (1)	3	1	MILD	MILD	YES	NO
RelA T505A 2	1	1	Yes (1)	2	0	MILD	MILD	YES	NO
RelA T505A 3	0	2	Yes (2)	2	0	MILD	MILD	YES	NO
RelA T505A 4	0	1	Yes (1)	1	0	MILD	MILD	YES	NO
RelA T505A 5	1	2	Yes (2)	3	0	NO	MILD	YES	YES
RelA T505A 6	0	5	NO	5	0	MODERATE	MILD	YES	NO
RelA T505A 7	1	7	Yes (3)	8	0	MILD	MILD	YES	NO
RelA T505A 8	0	3	Yes (1)	3	0	MODERATE	MILD	YES	NO
RelA T505A 9	2	0	NO	2	1	NO	MILD	YES	NO
RelA T505A 10	3	0	NO	3	0	MILD	MILD	YES	NO
RelA T505A 11	2	2	Yes (2)	4	0	MILD	MILD	YES	NO
RelA T505A 12	0	4	Yes (1)	4	1	MODERATE	MILD	YES	NO
