# Supplementary material for: A RelA(p65) Thr505 phospho-site mutation reveals an important mechanism regulating NF-κB-dependent liver regeneration and cancer
Source: Oncogene. 2016 Feb 8;35(35):4623–32. doi: 10.1038/onc.2015.526 (PMC4862573; doi:10.1038/onc.2015.526)
Supplement: Supplementary Information [file onc2015526x2.docx]

**A RelA(p65) Thr505 phospho-site mutation reveals an important mechanism regulating NF-κB dependent liver regeneration and cancer**

Anna Moles, Jacqueline A Butterworth, Ana Sanchez, Jill E Hunter, Jack Leslie, Helene Sellier, Dina Tiniakos, Simon J. Cockell, Derek A. Mann, Fiona Oakley and Neil D Perkins

**Supporting Figure Legends**

**Fig. S1. The phenotype of RelA T505A mice**

(A & B) Percentage organ:body weight of (A) heart and (B) right kidney from WT, WT/T505A and T505A twelve-week old littermate mice. WT, n = 4 males, 8 females; WT/T505A n = 2 males, 5 females; T505A n = 4 males, 5 females. NS: not significant.

(C) RT-PCR analysis showing equivalent levels of RelA transcripts in WT and RelA T505A mice.

(D) Representative confocal microscopy pictures of RelA nuclear translocation in WT and RelA T505A formalin fixed hepatocytes. Hepatocytes were cultured in 0% FBS media and treated with 50ng/mL of TNF-α for 10 or 30 minutes. This experiment was is representative of data from 3 separate hepatocyte preparations. A 20µm scale bar is shown.

**Fig. S2. Increased hepatocyte proliferation in RelA T505A mice following partial hepatectomy.**

(A & B) Mean number of BrdU-positive and HNE-positive cells/field in WT and T505A liver tissue sections following PhX surgery. Representative images of BrdU staining are shown. Photomicrographs are at x200 magnification.

(C) Gene Set Enrichment Analysis (GSEA) of microarray data from livers of WT and RelA T505A mice 36 hours following partial hepatectomy. Shown are the Enrichment plots for KEGG defined pathways for genes regulating cell cycle (KEGG_CELL_CYCLE) and homologous recombination (KEGG _HOMOLOGOUS_RECOMBINATION). The black vertical lines on these plots are, left to right in the same order as the genes top to bottom in the heat maps in Figure 2F.

(D) GSEA heat maps derived from the microarray data from livers of WT and RelA T505A mice 36 hours following partial hepatectomy. Shown are the results for KEGG defined pathways for genes regulating nucleotide excision repair (KEGG_NUCLEOTIDE_EXCISION_REPAIR; Enrichment Score (ES) -0.5064785; Normalized Enrichment Score (NES) -1.5508516; Nominal p-value 0.015686275; FDR q-value 0.08308681; FWER p-Value 0.704), mismatch repair (KEGG_MISMATCH_REPAIR; ES -0.67169833; NES -1.7920038; Nominal p-value 0.0019011407; FDR q-value 0.011143654; FWER p-Value 0.073) and DNA-replication KEGG_DNA_REPLICATION; ES -0.5400315; NES -1.6142532; Nominal p-value 0.01532567; FDR q-value 0.05392794; FWER p-Value 0.49).

**Fig. S3 Increased hepatocyte proliferation in RelA T505A mice following acute CCl4 administration.**

(A) Percentage liver:body weight at 24h, 48h and 72h post-partial acute CCl4 administration in male WT and T505A mice. (B & C) Mean number of αSMA-positive and BrdU-positive cells in WT and T505A liver tissue sections following PhX surgery. Representative images of BrdU staining are shown. Photomicrographs are at x200 magnification.

**Fig. S4 Increased hepatocyte proliferation and reduced fibrosis in RelA T505A mice following chronic CCl4 administration**.

(A) Percentage liver:body weight after 8 weeks of chronic CCl_4_ administration in male WT and T505A mice. (B) Serum transaminase, alanine aminotransferase (ALT) measurements in Arbitrary Units/Litre (U/L)

**Fig. S5. Early and increased onset of hepatocellular carcinoma in RelA T505A mice following DEN administration.**

(A) Representative images of PCNA staining following 48 or 72 hours of acute DEN administration. Photomicrographs are at x200 magnification (B) Percentage liver:body weight at 24, 48 and 72 hours post acute DEN administration in male WT and T505A mice. (C) Serum transaminase (ALT) measurements in Arbitrary Units/Litre (U/L). (D) Representative photomicrographs of Haematoxylin and Eosin (H & E) stained liver sections from 30 week DEN injured WT and RelA T505A mice. The top panel shows adenoma and the bottom panel shows hepatocellular carcinoma. An expert pathologist scored all pathology. Blue arrows show hepatic fat accumulation and back arrows denote mitotic bodies. Photomicrographs are at x200 magnification and representative of two different WT and two different RelA T505A+/+- mice. Scale bars are 100 microns.

**Fig. S6 Analysis of serum cytokine levels in wild type and in RelA T505A mice following DEN administration.**

(A-I) Serum level of KC/GRO (CXCL1), IL-2, IL-4, IL-5, IL-6, IL-10, IFNγ and TNFα in mice from the DEN model assayed by multiplex ELISA.

(J) RT-qPCR data showing relative KC/GRO mRNA expression in tumour tissue from WT and T505A livers 30 weeks after DEN administration. Data represents mean ± SEM, each point is an individual mouse.

Fig S7 (A) Representative images of active Caspase 3 staining following chronic DEN administration, black arrows denote active caspase 3+ hepatocytes. Photomicrographs are at x400 magnification (B) Representative images of PCNA staining following chronic DEN administration, black arrows denote PCNA+ hepatocytes. Photomicrographs are at x200 magnification.

**Table T1 Pathological scoring of tumours from 30 week DEN injured WT and RelA T505A mice.**

Analysis of the pathology of tumours and background liver tissue from 30 week DEN injured WT and RelA T505A mice scored by an expert pathologist. The background liver of wild type mice following DEN exposure showed mild to moderate portal and acinar inflammation, acinar disarray, hepatocellular changes (nuclear hyperchromasia, karyomegaly, cytoplasmic eosinophilia, Mallory-Denk bodies), hepatocyte proliferation with a variable number of mitotic figures, and hepatocyte apoptosis. Mild to moderate macrovesicular or mixed steatosis without steatohepatitis was observed in 7/13 cases, and stage 2 (6/13) to stage 3 (7/13) fibrosis was seen. There was no cirrhosis development. The background liver of RelA T505A NF-κB knock-in mice showed less severe DEN-induced fibrotic injury (stage 2 fibrosis in all cases) but increased steatosis (mild to severe in 10/12 cases, macrovesicular or mixed type). **Steatosis grade 1-3**: 1=mild (5-33%), 2=moderate (34-66%), 3=severe=>66% of hepatocytes (macrovesicular) *=mixed micro and macrovesicular type **=steatohepatitis

**Fibrosis Stage 0-4 on SRFG**: 0=no fibrosis, 1=zone 3 sinusoidal fibrosis (Z3SF), stage 2: Z3SF+portal/periportal fibrosis, Stage 3: bridging fibrosis, stage 4: cirrhosis

**Acinar Inflammation**: 1=0-1 focus/x10PF, 2=2-4 foci/x10PF, 3=>5 foci/x10PF

**Portal inflammation**: 0=absent, 1=mild, 2=moderate, 3=severe

**Hepatocyte injury** (incl. ballooning, nuclear pleomorphism and enlargement, eosinophilic cytoplasm,+/- Mallory-Denk bodies (MDB),+/- acinar disarray): 0=absent, 1=mild (few injured hepatocytes), 2: prominent (many injured hepatocytes)

**Apoptotic bodies**, **Mitoses**: 0=No, 1=yes

**HCC differentiation**: 1=well, 2=moderate, 3=poor
